# Editorial

**Published:** 1903-07-15

**Authors:** 


### EDITORIAL.

By reference to the article represented from the *Dental Record* in this number it will be seen that our Scotch brothers are agitating the question of a higher learning for dentists. There seems to run through the discussion a conviction that the technical aspect of the present licentiate of dentistry is not what it should be. This may be more apparent than real, for it is a criticism easily made by the experienced practitioner of the most accomplished college graduate. We sometimes think too much is expected of college men. But the burden of this discussion seems to be that a post graduate course, embracing a more exacting study of the related sciences and art principals of dentistry should be rewarded with an academic insignia, which should stand in the public estimation for a broader culture than is usual or necessary to gain a permit to practice this special healing art. It will also be noted that the prevalent practice of seeking this preferment in the general medical title is discredited.

That this matter has come up for discussion in this particular country is not strange, since it seems to be the one topic of greatest interest in all gatherings of dentists interested in educational affairs. It may not always assume this particular line of discussion. There seems to be almost a unanimous opinion that a sufficient number of skillful techniques has been developed to care for the public needs, and that the time has now come for the training of professional men with not only higher mental and technical attainments but with capacities for scientifically studying the many perplexing problems involved in the intelligent administration of a professional career in so narrow a field as the practice of dentistry. In this country it has not taken the form of a university degree, but just now is expressing itself in the efforts to perfect the curriculum best adapted for practical administration by our schools with their present or possible equipment. This subject will doubtless occupy much of the time of the National Faculties Association this summer, and the action to be taken there will mean much as to what position the profession in this country will take on the matter of higher education of the dentist. Some favor higher preliminary training, others a higher development of the technical features, and some a broader and better preparation for scientific research, while, in some places, there seems to be a desire to develop a kind of professional aristocracy. We believe the general motive is to stimulate higher education for its own sake, and that a more skillful profession shall command the public esteem and honor which satisfies the longings for compensatory rewards other than pecuniary. Whatever the motive there evidently is a strong sentiment for an advance in educational standards.

